# Recent advances in understanding Cushing disease: resistance to glucocorticoid negative feedback and somatic USP8 mutations

**DOI:** 10.12688/f1000research.10968.1

**Published:** 2017-05-02

**Authors:** Eleni Daniel, John Newell-Price

**Affiliations:** 1 Department of Oncology and Metabolism, University of Sheffield Medical School, Sheffield, UK

**Keywords:** pituitary corticotroph adenoma, adrenocorticotropic hormone, cortisol

## Abstract

Cushing’s disease is a rare disease with a characteristic phenotype due to significant hypercortisolism driven by over-secretion of adrenocorticotropic hormone and to high morbidity and mortality if untreated. It is caused by a corticotroph adenoma of the pituitary, but the exact mechanisms leading to tumorigenesis are not clear. Recent advances in molecular biology such as the discovery of somatic mutations of the ubiquitin-specific peptidase 8 ( *USP8*) gene allow new insights into the pathogenesis, which could be translated into exciting and much-needed therapeutic applications.

## Introduction

Cushing’s disease (CD) is caused by a pituitary corticotroph adenoma over-secreting adrenocorticotropic hormone (ACTH) leading to excess cortisol secretion ^1^. It is a rare disease, associated with high mortality and morbidity if untreated ^2^. Morbidity is mainly due to the chronic metabolic adverse effects of hypercortisolism leading to the clinical features that include those due to protein wasting (skin thinning, myopathy), changes in fat distribution and glucose intolerance, osteoporosis, life-threatening infections, and psychiatric and cognitive changes, including depression and psychosis ^3^. More than 90% of corticotroph adenomas present as microadenomas (that is, small tumors with a maximum diameter of less than 10 mm), but occasionally tumors can be macroadenomas of significant volume and cause pressure to surrounding structures such as the optic chiasm and the cavernous sinuses and may have invasive features. The only curative treatment is surgical resection of the tumor, usually performed through a transsphenoidal approach. Although initial remission is achieved in around 60% to 75% of patients, up to 30% experience recurrent disease on long-term follow-up. The treatment of recurrent disease is challenging, and options include radiotherapy, medical therapy, or additional surgery ^4^.

The pathogenesis of corticotroph adenomas is not clear. Corticotroph adenomas are monoclonal in origin, meaning that they arise from a single cell that multiplies to cause tumor growth ^5– 8^. This strongly points to a single somatic genetic defect in a corticotroph cell as the etiologic mechanism of disease ^5^ and implies that tumorigenesis occurs at the level of the pituitary. However, it is possible that other hypothalamic factors allow or facilitate this process. Genetic mutations have been identified in corticotroph adenomas; however, we are far from completely understanding the mechanisms leading to tumorigenesis, ACTH hyper-secretion, and invasiveness.

A better understanding of the pathogenesis could help identify new therapeutic targets. Existing medical treatments aim to control the hypercortisolemia associated with this condition and its devastating long-term effects but are not always effective. In this report, we present the current understanding of the pathogenesis of CD with an emphasis on molecular discoveries that have been reported in the last few years. These discoveries have created new possibilities for therapeutic targets, much needed for patients who cannot be cured by surgery. We will provide an overview of corticotroph tumorigenesis in the context of hypothalamic-pituitary-adrenal (HPA) axis regulation with an emphasis on the role of the glucocorticoid receptor in the resistance to the negative feedback of cortisol that occurs in CD, and we will explore the role of epidermal growth factor receptor (EGFR) signaling in ACTH hyper-secretion and corticotroph cell proliferation and the recent discovery of somatic ubiquitin-specific peptidase 8 (USP8) mutations in a significant number of patients with sporadic CD with an emphasis on therapeutic implications.

## Corticotroph adenoma pathogenesis and the hypothalamus-pituitary-adrenal axis

### Normal physiology

Corticotroph cell function is tightly regulated as part of the HPA axis. Hypothalamic corticotropin-releasing hormone (CRH) and arginine vasopressin (AVP) stimulate pituitary corticotroph cells to secrete ACTH ^9^. CRH acts by binding to a G protein-coupled receptor on the cell surface of the corticotroph cells, CRH-R1. Ligand binding to CRH-R1 activates stimulatory G-protein alpha subunit (G _as_), causing downstream intracellular signaling through cAMP and protein kinase A ^10, 11^. The final event of the signaling cascade is promotion of proopiomelanocortin gene ( *POMC*) transcription and ACTH release ^11, 12^. POMC is a precursor polypeptide and upon cleavage produces ACTH ^13^. In turn, ACTH binds to melanocortin 2 receptor (MC2R) on the surface of adrenocortical cells and stimulates the steroidogenesis pathway. The end product, cortisol, is released to the circulation and regulates the HPA axis by negative feedback through the glucocorticoid receptor type 2 (NR3C1) at the level of the hypothalamus and the pituitary. The NR3C1/cortisol complex translocates from the cytoplasm to the nucleus to bind to regulatory areas on the DNA and inhibits synthesis of POMC, CRH, and AVP mRNA and therefore reduces ACTH secretion. In theory, deregulation of any part of this complex process at the level of hypothalamus, pituitary, or negative glucocorticoid feedback mediated by NR3C1 could lead to tumorigenesis.

### The role of cortisol-negative feedback in corticotroph tumorigenesis

Loss of sensitivity to the negative cortisol feedback at the level of the pituitary and hypothalamus is a key feature of CD and is used for biochemical diagnosis ^14^. Normally, *in vivo* administration of an exogenous glucocorticoid such as dexamethasone leads to suppression of endogenous cortisol and ACTH production, whereas in CD there is inadequate suppression of cortisol levels ^3^. Incubation of primary cultures from corticotroph adenomas with cortisol causes a reduction in ACTH levels, indicating some response to negative feedback ^15^; however, dexamethasone treatment causes less inhibition of ACTH release and POMC mRNA levels in cultured corticotroph adenoma cells than non-adenomatous pituitary cells ^16^. Downregulation of the glucocorticoid receptor or mutations in its signaling pathway could be a plausible explanation of glucocorticoid resistance; however, in ACTH-producing corticotroph adenomas, the expression of the receptor has been found to be increased, and although NR3C1 mutations have been found in cases of CD, these are not frequent ^17, 18^. At the pre-receptor level, 11β-hydroxysteroid dehydrogenase type 2, a key enzyme that regulates cortisol activity in the tissues by converting active cortisol to inactive cortisone, may also be involved as it has been found to be highly expressed in corticotrophinoma cells but not normal corticotroph cells, indicating a mechanism through which the feedback of cortisol to the pituitary could be compromised in tumor cells ^19, 20^. Recent studies elucidate other mechanisms through which NR3C1 is implicated in the resistance to the negative glucocorticoid feedback seen in CD.

### Testicular orphan nuclear receptor 4

Testicular orphan nuclear receptor 4 (TR4) is a nuclear receptor encoded by the NR2C2 gene and acts as a regulator of transcription (activator or repressor) in various tissues, including the central nervous system and reproductive tissues ^21^. A murine knockout for *TR4* exhibits growth retardation, weight loss, and reduced lipid accumulation ^22– 24^. TR4 is overexpressed in corticotroph adenomas and corticotroph tumor cell lines and activates *POMC* by binding to its promoter, an effect that is enhanced by phosphorylation of TR4 through the mitogen-activated protein kinase/extracellular signal-regulated kinase (MAPK/ERK) pathway. Overexpression of TR4 also induces ACTH secretion, cell proliferation, and tumor growth in a murine animal model harboring ACTH-secreting tumors ^25^. Further studies showed that TR4 interacts with the glucocorticoid receptor, NR3C1, and overrides the negative regulation of NR3C1 on *POMC* transcription and ACTH secretion ^26^. This indicates a pathway through which TR4 promotes resistance to the negative glucocorticoid feedback. Previous studies have shown that TR4 can be trans-activated by peroxisome proliferator-activated receptor-gamma (PPARγ) agonists such as rosiglitazone and polyunsaturated fatty acids ^22^. In contrast to the mechanism described above, rosiglitazone has been shown to reduce ACTH secretion in cell lines, but clinical trials in patients with CD have had mixed results ^27– 30^. A possible explanation for this is that PPARγ agonists trigger multiple pathways in corticotroph cells, and further research into the role of TR4 may help identify other specific therapeutic targets for the treatment of CD.

### Heat shock protein 90

The *in vivo* function of the glucocorticoid receptor is heavily dependent on its interaction with the heat shock protein 90 (HSP90). HSP90 is a chaperone protein that stabilizes and activates proteins through induction of conformational changes. HSP90 protein interacts with the glucocorticoid receptor to facilitate ligand binding and aids its translocation to the nucleus, where NR3C1 binds to DNA and promotes transcription ^31, 32^. Corticotroph adenomas overexpress HSP90, and some inhibitors of HSP90 can enhance the transcriptional activity of the glucocorticoid receptor by inducing its release from HSP90 in a stable and high-affinity state for ligand binding ^33^. Silibinin, a C-terminal HSP90 inhibitor, increased the transcriptional activity of NR3C1 in murine corticotroph cells and enhanced the suppression of ACTH secretion in primary corticotroph adenoma cell cultures, restoring glucocorticoid sensitivity *in vitro*. In keeping with the *in vitro* data, oral administration of silibinin in a murine CD model caused reductions in clinical features and tumor growth and in ACTH and endogenous glucocorticoid levels. Silibinin is a commercially available extract of milk thistle seeds and has been used in the treatment of prostate cancer and hepatotoxicity with a good safety profile and therefore is an interesting agent for assessment in the treatment of CD ^34^, and clinical trials using this agent have been proposed.

## Familial endocrine syndromes and Cushing’s disease

Corticotroph adenomas are usually sporadic and only rarely have been observed as part of familial endocrine genetic syndromes. Germline mutations that cause familial endocrine syndromes affect different pathways, are seen infrequently in CD, and do not explain the majority of the corticotroph adenomas or indicate a common mechanistic explanation for the pathogenesis of CD. Familial CD has been reported in germline mutations of the tumor suppressor *MENIN* (causing multiple endocrine neoplasia type 1 syndrome, or MEN1), aryl-hydrocarbon receptor-interacting protein gene ( *AIP*), and *CDKN1B* gene (or *p27/Kip1*) that encodes for p27, a cell cycle inhibitor and causes multiple endocrine neoplasia type 4 (MEN4) ^35– 41^. McCune-Albright syndrome is caused by post-zygotic somatic mutations in the *GNAS1* gene that encodes a stimulatory G _as_ and causes a constitutive activation of the Gsα-cAMP signaling pathway. The disease is commonly associated with somatotrophinomas, and only three cases of CD have been described as harboring *GNAS* mutations in the tumor tissue ^35, 42, 43^.

## EGFR signaling

EGFR is a cell surface receptor with tyrosine kinase activity and is a member of the ErbB family of cell surface tyrosine kinase receptors. It is activated by ligand binding with peptide growth factors such as epidermal growth factor (EGF) ^44^. Upon ligand binding, it forms homodimers, and the intracellular tyrosine kinase domain is activated. This promotes signal transduction through complex downstream phosphorylation pathways involving the MAPK pathway, the phosphoinositol kinase (PI3), phospholipase C gamma (PLCγ), and transcription factors, culminating in promoting cell proliferation and cell differentiation in various tissues ^45– 47^. Following signal transduction, the ligand-activated EGFR internalizes and is tagged with ubiquitin protein and degraded in the lysosomes ^48^. EGFR signaling confers a powerful proliferation signal; overexpression of EGFR has been found in several cancers, and EGFR inhibitors are used in the treatment of these tumors ^49– 51^.

EGFR signaling promotes proliferation and ACTH secretion in corticotroph cells. EGFR and its ligand EGF are highly expressed in corticotroph adenoma cells in up to 75% of corticotroph tumors as well as normal pituitary cells, gonadotroph, somatotroph, and lactotroph adenoma cells ^52– 55^. Higher levels of expression of EGFR/EGF in corticotroph adenoma cells are correlated with more aggressive tumors ^52, 54, 56– 58^. EGFR signaling in corticotroph cells could lead to proliferation by downregulation of *p27/Kip1* through a MAPK/ERK pathway ^55^. Low expression of p27 has been found in CD, and a mouse knockout model for *p27/kip1* gene develops corticotroph tumors of the intermediate lobe and weight gain ^59– 61^. In humans, however, no somatic mutations of *p27/kip1* were found in 20 corticotroph adenomas ^62^. Additionally to its proliferating effects, EGFR signaling promotes *POMC* expression and ACTH secretion through activation of the MAPK pathway ^15, 57, 63^. In contrast, inhibition of EGFR signaling by gefitinib, an EGFR kinase inhibitor, inhibits *pomc* expression in mice and corticotroph cell proliferation in cell cultures, decreasing tumor growth and cortisol levels with improvement of clinical features ^64^.

## Somatic USP8 mutations in corticotroph adenomas

Sporadic corticotroph adenomas only rarely harbor somatic mutations in the genes that cause CD by germline mutations ^65^. Recently, extremely elegant whole-exome sequencing and functional studies have shown that somatic mutations involving the *USP8* gene are found in 35% to 62% of sporadic corticotroph adenomas, providing significant insight into the mechanisms of disease and a direct link with EGFR signaling ^66^. *USP8* is located on chromosome 15q21.2 and encodes a deubiquitinating enzyme, a protein member of the ubiquitin-specific processing protease family ^67^. Ubiquitination is a reversible post-translational modification that targets proteins, including cell surface receptors, for degradation by the endosome-lysosome system through conjugation with a single or multiple ubiquitin proteins at lysine residues ^68^. USP8 catalyzes the cleavage of ubiquitin tags (deubiquitination) and is involved in tyrosine kinase receptor trafficking and endosome-lysosome function, leading to receptor recycling to the cell surface ^69– 72^.

Further studies confirmed these findings; somatic *USP8* mutations were found in 35% of patients with CD (21 out of 60) in a series from Japan, 62% of corticotroph adenomas (75 out of 120) in a large series from China, and 36% (48 out of 134) in an international series ^73– 75^. These mutations seem to be specific to corticotroph adenomas since no *USP8* mutations were detected in 80 non-functioning pituitary adenomas, 80 prolactinomas, 84 growth hormone-secreting adenomas, and 58 pituitary adenomas of other etiologies ^73, 75, 76^. USP8 has a 14-3-3 protein-binding site, and the *USP8* mutations found in corticotroph adenomas clustered to the 14-3-3 protein-binding motif encoded by exon 14 of the USP8 gene. 14-3-3 proteins are highly conserved regulatory proteins that bind to common recognition motifs and modify protein activity and interactions of the protein with other molecules; in the case of USP8, binding of 14-3-3 protein inhibits its deubiquitinating activity ^77– 79^. The *USP8* mutations found in CD caused impaired 14-3-3 protein binding which, in the majority of the mutations described, resulted in a proteolytic cleavage immediately upstream the 14-3-3 binding site because of an unidentified protease accessing the site. The cleavage created two fragments of USP8 protein, sized 40 and 90 KDa; the 40-KDa fragment possessed increased deubiquitinating activity and caused a significant inhibition of EGFR downregulation by degradation at the lysosomes, increased re-cycling of EGFR, and augmented EGFR-induced MAPK signaling leading to high POMC mRNA expression ^66, 74, 75, 80^.

In corticotroph adenomas, *USP8* mutations were more likely in females, smaller-sized tumors, and microadenomas ^66, 74, 75, 80^. No difference in age, serum cortisol secretion, or Ki67 index was reported ^74^. An international multicenter study in 134 secreting and 11 silent corticotroph adenomas in 105 adults and 29 pediatric cases showed that 36% of secreting corticotroph adenomas (48 out of 134) carry *USP8* mutations but that none of the silent corticotroph adenomas harbored mutations and that tumors carrying *USP8* mutations were more likely in adults ^80^.

These findings have clear implications that may translate into much-needed therapeutic applications since the identified signaling pathways could be targeted for treatment of CD. Inhibition of USP8, EGFR, or downstream signaling regulators and molecules involved in these pathways holds promise for treating *USP8*-mutated disease. In primary corticotroph cell cultures consisting of *USP8*-mutated tumor cells, USP8 small interfering RNA (siRNA) knockdown or blocking of EGFR attenuated ACTH secretion, an effect that was also achieved by treatment with the currently available EGFR inhibitor, gefitinib ^75^. Furthermore, molecular characterization of tumors may help inform drug selection as *USP8*-mutated corticotroph tumors were more likely to express somatostatin receptor 5 (SSTR5), a receptor that can be targeted by the somatostatin analog pasireotide, and O6-methylguanine–DNA methyltransferase ** ( *MGMT*) mRNA, indicating less favorable response to temozolomide, an alkylating chemotherapy agent used in aggressive CD ^74^. More studies are needed to confirm these findings. However, clinical applications are already emerging; the EGFR inhibitor gefitinib is currently being assessed for the treatment of hypercortisolemia in CD in a phase 2 study in China ^81^.

## Conclusions

Understanding the mechanisms leading to the development of CD has been restricted by the low incidence of disease and limited tissue availability for research. Recent molecular developments give intriguing insights into the pathogenesis of CD in a significant number of corticotroph tumors. However, we are still far from understanding the neoplastic process completely. There has been progress in understanding the mechanism of glucocorticoid feedback resistance, a central feature in CD that allows tumors to escape the physiological regulatory mechanisms, through the identification of the interaction of the glucocorticoid receptor with transcription regulators TR4 and HSP90. The discovery of *USP8* mutations in a significant number of corticotroph adenomas (35% to 62%) highlighted the role of enhanced EGFR signaling in the pathogenesis of CD; untangling the interactions of downstream signaling molecules in this pathway (or these pathways) opens up a new area of research into CD pathogenesis. Molecules involved in USP8/EGFR and TR4 signaling pathways as well as selective inhibitors of HSP90 are emerging as attractive therapeutic targets, especially as no ideal treatment exists for treating corticotroph adenomas not cured by surgery, possibly paving the way for personalized medicine in the future. The outcomes of clinical trials using compounds that target these pathways are keenly awaited.

## Abbreviations

ACTH, adrenocorticotropic hormone; AVP, arginine vasopressin; CD, Cushing’s disease; CDKN1B, cyclin-dependent kinase inhibitor 1B; CRH, corticotropin-releasing hormone; CRH-R1, corticotropin-releasing hormone receptor 1; EGF, epidermal growth factor; EGFR, epidermal growth factor receptor; ERK, extracellular signal-regulated kinase; HPA, hypothalamic-pituitary-adrenal; HSP90, heat shock protein 90; MAPK, mitogen-activated protein kinase; NR3C1, glucocorticoid receptor; POMC, proopiomelanocortin; PPARγ, peroxisome proliferator-activated receptor-gamma; TR4, testicular orphan nuclear receptor 4; USP8, ubiquitin-specific peptidase 8.
